# Seasonal levels and size fractions of indoor air bioaerosols as predictors of respiratory morbidities among school pupils in Ibadan, Nigeria

**DOI:** 10.3389/fpubh.2025.1611167

**Published:** 2025-10-03

**Authors:** Oyewale Mayowa Morakinyo, Godson R. E. E. Ana

**Affiliations:** Department of Environmental Health Sciences, Faculty of Public Health, College of Medicine, University of Ibadan, Ibadan, Nigeria

**Keywords:** indoor air quality, microbial aerosols, aerosol size distribution, public primary school, Ibadan

## Abstract

**Introduction:**

Bioaerosols are among pollutants that impair indoor air quality in schools and have been associated with increased respiratory morbidities. Knowledge of bioaerosols’ sizes and the likely deposition sites within the child’s respiratory tract are essential for the identification of associated risks. This study was designed to determine bioaerosols’ size distribution and associated respiratory morbidities among school pupils in Ibadan North Local Government Area (INLGA), Ibadan.

**Methods:**

A descriptive cross-sectional study design was adopted. In nine randomly selected public primary schools in INLGA, indoor air sampling (when occupied and unoccupied) was conducted thrice weekly for 3 months each in the wet and dry seasons, respectively. Airborne Bacterial Respirable Fraction (BRF) and Fungal Respirable Fraction (FRF) of aerodynamic diameter of 1.1–4.7 μm which corresponds to regions between the human primary bronchus and the alveolar duct were sampled using a six-stage cascade impactor. The BRF and FRF were estimated and dichotomised into high (>median) and low (≤median) categories. A standardized questionnaire was adapted to elicit information from 554 randomly selected pupils on socio-demographic characteristics and self-reported respiratory morbidities. Data were analyzed using descriptive and inferential statistics at α_0.05_.

**Results:**

Median BRF and FRF during the wet season (2,890 and 283 cfu/m^3^) were significantly higher than dry season (1,661 and 196 cfu/m^3^), respectively and above WHO standards. Median BRF and FRF were significantly higher when classrooms were occupied (3,906 and 230 cfu/m^3^) than unoccupied (2,800 and 214 cfu/m^3^), respectively. About 67.5% of total bacterial and 77.8% fungal aerosols were respirable fractions. Age of pupils was 10.8 ± 1.35 years and 57.4% were males. Exposure to high BRF and FRF was significantly associated with current rhinitis (aOR = 1.78, 95%CI: 1.11–2.85 and aOR = 1.83, 95%CI: 1.14–2.93) and current wheeze (aOR = 2.77, 95%CI: 1.73–4.43 and aOR = 1.88, 95%CI: 1.18–3.00), respectively. Male pupils were more likely to experience current rhinitis (aOR = 1.09, 95%CI: 1.15–1.58) and current wheeze (aOR = 1.11, 95%CI: 1.22–1.62) than females.

**Conclusion:**

Exposure to high levels of respirable bacterial and fungal fractions was associated with respiratory health outcomes among pupils.

## 1 Introduction

Clean air is a basic human right as well as a necessity for life and good health. Indoor air quality (IAQ) refers to the characteristics of the air in indoor settings where people spend a substantial amount of time each day, such as homes, workplaces, schools, and other built settings. It is a determining factor in ensuring a healthy life and people’s wellbeing (1).

Children spend a significant amount of time indoors, both at home and at school, with the latter being the second most common indoor environment after residences (2). The hours spent in school may expose them to potentially harmful pollutants (3). Children are at a greater risk of being harmed from exposure to indoor pollution than adults since they have smaller airways and underdeveloped respiratory and immunological systems (4). They also breathe in more air per unit mass than adults. While exposures may be small, they can accumulate and, in combination, have the potential to contribute to adverse health conditions (4).

Indoor air in schools is known to harbor bioaerosols (5). Bioaerosols are microbial fractions of solid and liquid particles (6). They are a mix of living (bacteria, fungi, viruses) and non-living microorganisms (endotoxins, metabolites, toxins) dispersed in the air, ranging in diameter from 0.5 to 30 m (7). They represent about 5–10% of airborne particulate matter and are universally present in the environment (8). The ability of bioaerosols to cause disease is determined by the amount of inhaled bioaerosols and their sizes, which dictate the site of deposition on human respiratory systems (9). For instance, inhalable bioaerosols usually settles in the extra thoracic region of humans while respirable bioaerosols can get to the regions of the trachea, bronchial and alveolar (10).

The outcomes of exposure to bioaerosols vary by season and constitute a public health challenge (11). Bioaerosols in indoor air can induce allergies and may be injurious to health (12). A significant population of fungi and bacteria in the indoor environment can cause mucous membrane irritation, fatigue, headaches, memory loss, and newborn bronchiolitis. (13). Studies have revealed that poor IAQ resulted in more illnesses, absenteeism, asthma attacks (9), respiratory and cardiopulmonary pathologies (14).

To lower the incidence of respiratory illnesses among Nigerian children, scientific and evidenced-based findings are needed to understand IAQ in primary schools. There is insufficient data on the types and possible sites of deposition of respirable bioaerosols within the respiratory tract of school children based on their size fractions. Having an understanding of the association between the levels and size fractions of bioaerosols, deposition sites within the respiratory tract and inhalation dose of bioaerosols in the indoor school environment is crucial to instituting appropriate strategies to protecting the vulnerable populations from poor IAQ.

This study sought to (i) quantify the seasonal burden and characteristics of indoor bioaerosols responsible for the reported respiratory morbidities among school children, and (ii) determine the levels, size distributions and deposition sites of bacterial and fungal particles.

## 2 Methodology

### 2.1 Study area

**Figure 1:**
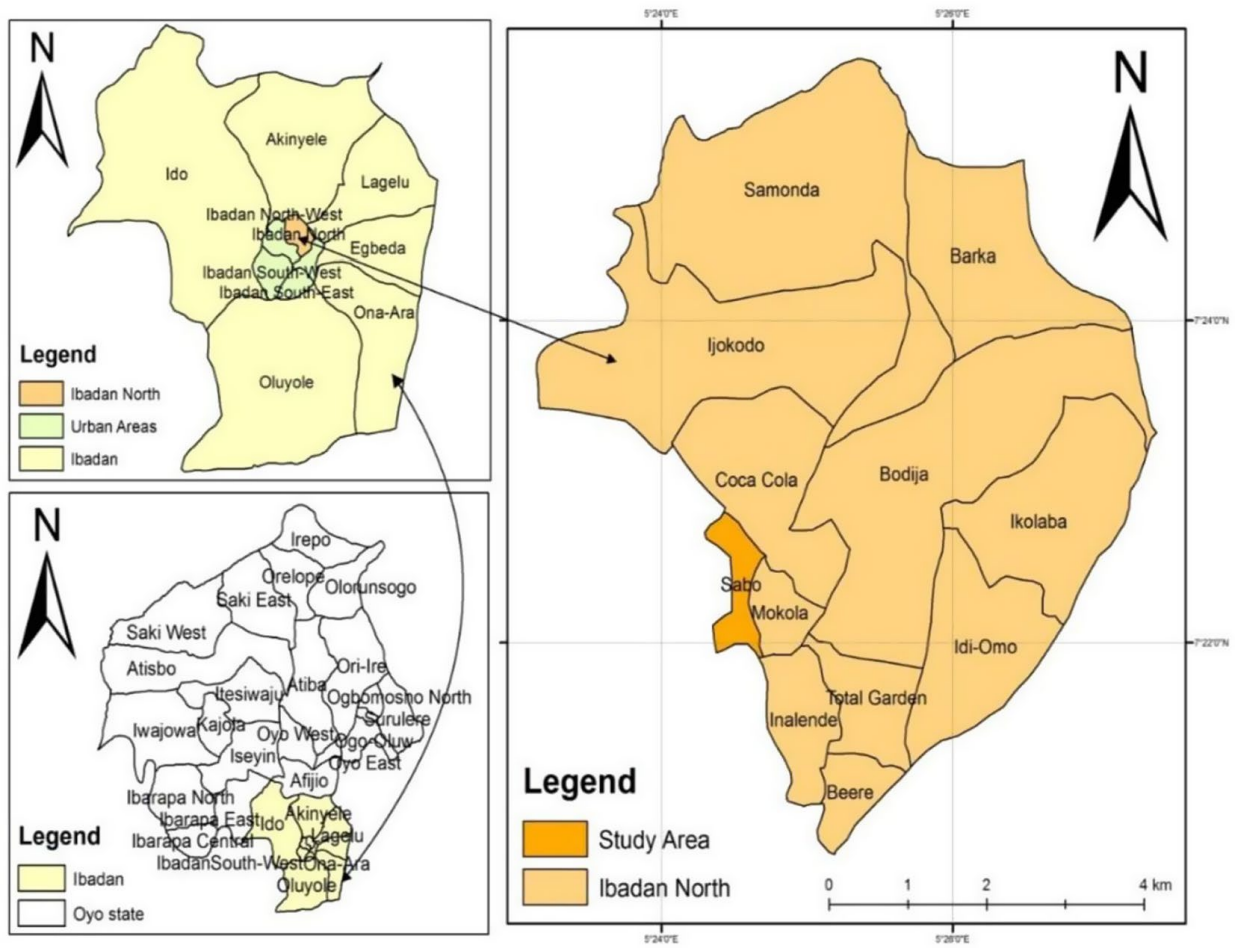
Map of Ibadan North Local Government Area.

This study was carried out in Ibadan, the capital of Oyo State in western Nigeria. Ibadan is located on longitude 7°23′47″N, latitude 3°55′0″E and at an altitude of 152–213 m (15). It has an estimated population of 4,144,130 (16). Ibadan’s climate is tropical, with distinct wet and dry seasons and a mean minimum annual temperature of 210°C. The city has 11 Local Government Areas with a total area of 103.8 sq. km (LGAs). Ibadan North LGA (Figure 1) is one of the 11 LGAs where the study was conducted.

### 2.2 Study design

A mixed study design was adopted involving field and laboratory components comprising questionnaire administration, indoor and outdoor air quality monitoring for bioaerosols’ size distribution and deposition.

### 2.3 Study population

The study population were primary school pupils in classes three to six (Grade 3–6) in the selected schools. An eligible study participant must have attended the school for at least 1 year. Study participants were selected using a systematic random sampling. A total of 520 consented pupils were recruited into the study.

### 2.4 Selection of schools and sampling site

One-stage sampling method that involves balloting was used in randomly selecting Ibadan North Local Government Area (INLGA) from the 11 LGAs in Ibadan. Also, balloting was used to select a total of nine public primary (mixed schools) schools in INLGA. The selection of classrooms for the evaluation of the seasonal fluctuations and size distribution of indoor bioaerosols was based on key environmental and building features such as building types, building materials, ventilation type, etc. Also, schools with comparable student populations and classroom occupancy rates were given preference in order to reduce selection bias and improve comparability across study sites.

### 2.5 Data collection methods

Data collection for this study was carried out in three phases:

- Air sampling of viable airborne bioaerosols into size fractions using a six-stage microbial impactor.
- Estimation of dose rates of respirable bacterial and fungal aerosols.
- Determination of the prevalence of reported respiratory symptoms and other characteristics using a semi-structured questionnaire.

### 2.6 Instrument for bioaerosols’ sampling

A six-stage Honri Airclean (Model FSC-A6) impactor Air Sampler was deployed for sampling of bacterial and fungal aerosols into various sizes. It was designed such that particles of different sizes are aerodynamically sized and compared with the human respiratory tract. As a result, the sampler can estimate the disease-causing potential of airborne particles based on their deposition site within the respiratory tract. The aerodynamic diameters of the six-stage impactor are >7 m (stage 1), 4.7–7 m (stage 2), 3.3–4.7 m (stage 3), 2.1–3.3 m (stage 4), 1.1–2.1 m (stage 5), 0.65–1.1 m (stage 6) corresponding to the surface, pharynx, trachea, and primary, secondary, terminal, and alveoli regions of the human respiratory tract, respectively (17). The impactor allowed for a uniform bioaerosol sampling methodology, which ensured a consistent sampling of bioaerosols across respirable and non-respirable particle fractions. This method allowed size-segregated bioaerosol profiles under various seasonal conditions to be robustly compared.

### 2.7 Sampling of bioaerosols and culture medium

The field sampling for airborne bacteria and fungi was carried out in nine schools in both outdoor and indoor when the classrooms were unoccupied and occupied. Sampling was done thrice weekly for 3 months each in the wet and dry seasons. Given the impact of meteorological conditions including humidity, rainfall, and temperature, sampling was done both in the wet and dry seasons to accurately capture seasonal fluctuation in bioaerosol concentrations and types. To guarantee equal representation of both seasons in all chosen schools, sample collection was scheduled.

The sampling protocol previously used by Fang et al. (18, 19) was employed in this study. Prior to sampling, each of the six stages of the FSC-A6 microbial sampler were sterilized with 75% alcohol and loaded aseptically (inside a Class II biosafety cabinet) with 90 mm Petri dishes containing MacConkey agar medium and Sabouraud Dextrose Agar medium (contained a 100 mg of chloramphenicol to inhibit bacterial growth) after its sterilization with a 75% alcohol (19) for bacterial and fungal sampling, respectively.

The FSC-A6 microbiological sampler was set up on a platform of height 1.5 m at each sampling point in each school, aiming for the children’ breathing zone and operated at a flow rate of 28.3 L/min for 15 min during two sampling periods (8:00 am and 2:00 pm), respectively. A sampling time of 15 min was selected because according to Tortora et al. (20) and Jensen and Schafer (21), this is the approved sampling time for obtaining between 30 and 100 microbial cells on a single petri dish.

### 2.8 Estimation of microbial counts

The plates were transported in an icebox to the laboratory and incubated in an incubator for 48 h at 37°C for bacterial samples and 72 h at 25°C for fungal samples (17). To adjust for overlapping colonies, the number of bacterial and fungal colonies that developed was counted using the positive-hole correction technique (22).

Using the formula (Equation 1), the total bacterial count (TBC) and total fungal count (TFC) were calculated in colony-forming units per cubic meter (cfu/m^3^) (17):


(1) $$\begin{array}{l} \text{Total bacterial}/\\ \text{fungal count}\;(\mathrm{cfu}/m^{3})=\;\frac{\left[\begin{array}{l} \text{Total bacterial}/\\ \text{fungal colonies}\times 103 \end{array}\right]}{\left[\begin{array}{l} \mathrm{Air}\;\text{flow rate}\times \\ \text{time}\;(\text{minutes}) \end{array}\right]} \end{array}$$

The concentration of bacteria and fungi at each sampling point for each stage was estimated using Equations 2, 3 as follows:


(2) $$B_{\mathrm{ti}}=\frac{\mathrm{Ci}}{\mathrm{TBC}}$$

Where *B_ti_*: bacterial size fraction (%); *Ci*: the concentration of bacteria on stage *i*; TBC: Total bacteria concentration for all the stages.


(3) $$F_{\mathrm{ti}}=\frac{\mathrm{Ci}}{\mathrm{TFC}}$$

Where *F_ti_*: Fungal size fraction (%); *Ci*: the concentration of fungi on stage *i*; TFC: total concentration of fungi (23)The total Bacteria Respirable Fraction and the Total Fungi Respirable Fraction was estimated using Equations 4, 5 as follows:


(4) $$\text{Bacteria Respirable Fraction}\;(\mathrm{BRF})=\frac{C3+C4+C5+C6}{\mathrm{TBC}\times 100\%}$$

(5) $$\text{Fungi Respirable Fraction}\;(\mathrm{FRF})=\frac{C3+C4+C5+C6}{\mathrm{TFC}\times 100\%}$$

### 2.9 Questionnaire administration

A standardized questionnaire by the International study on Asthma and Allergies in Childhood (ISAAC), was adapted and administered on pupils in classes’ three to six to elicit information on socio-demographic characteristics of pupils, environmental conditions of classrooms, perceived health effects, frequency of occurrence of perceived health complaints and symptoms, associated with exposure to indoor air pollutants.

Moreover, another semi-structured questionnaire was used to elicit information from parents on: demographic characteristics, child’s history of respiratory infections, risk factors for respiratory infections, household population and household characteristics, substance use, etc.

### 2.10 Dependent and independent variables

The dependent variables include current rhinitis, rhinitis ever, current wheeze and wheeze ever. The definition of the health outcomes as stated in the questionnaire is presented in Table 1. The main predictors of the health outcomes in this study include respirable bacterial and fungal aerosols, and total bacterial and fungal aerosols. Other independent variables or covariates identified in this study include age (<10 or ≥10 years) and gender (male or female) of the pupil, and type of fuel used for cooking at home.

**Table 1:** Dependent variables.

Respiratory symptoms	Question
Current rhinitis	Have you experienced a problem with sneezing or a runny or clogged nose in the last 12 months when you were not sick with a cold or flu? (Yes/No)
Rhinitis ever	Have you ever had a problem with sneezing or runny or blocked nose, when you did not have a cold or flu? (Yes/No)
^*^Current wheeze	Has your child experienced chest wheezing or whistling in the recent 12 months? (Yes/No)
	How many attacks of wheezing has your child had in the last 12 months? *Pupils who experienced 4–12 or more than 12 attacks were considered positive for current wheeze.*
	How many times has your child’s sleep been disrupted by wheezing in the last 12 months? *Pupils who indicated one or more nights per week included in the definition of current wheeze*
Wheeze ever	Has your child ever had wheezing or whistling in the chest at any time in the past?

* To improve the specificity of the results, we decided to use a stricter definition of ≥4 wheezing episodes in the previous 12 months to define “current wheeze” in our study.

### 2.11 Data analysis

The data were analyzed using a non-parametric approach because they were not normally distributed. Thus, bacterial and fungal concentrations were described by median and interquartile range. In addition, to better depict the size distribution, geometric median diameter was derived. All statistical calculations, including univariate and multivariate analyses (which included the Pearson correlation coefficient, nonparametric Mann–Whitney U, and Wilcoxon matched pairs tests), were performed using the statistical program for social science (SPSS) version 25.0. The degree of correlation between variables was estimated using Chi square statistics and binary regression. The Mann–Whitney U test was employed to ascertain if bioaerosols’ concentrations varied across seasons and sampling location. The Wilcoxon test compared the size fractions of bioaerosols over different seasons (when classrooms were unoccupied and occupied).

The unadjusted and adjusted odds ratios and 95% confidence intervals were calculated to estimate the likelihood of having rhinitis ever, current rhinitis, wheeze ever, and current wheeze given the presence of a potential risk factor. Step by step, confounding or other independent variables were introduced, beginning with the most significant from the univariate analysis.

### 2.12 Ethical approval

The University of Ibadan and the University College Hospital Ethical Board (UI/UCH) Ethics Committee approved the study (Approval number: UI/EC/16/0045). The Oyo State Ministry of Education and the State Ministry of Health granted permission to conduct the research. The Boards and Management of the selected schools, as well as the Parents’ and Teachers’ Association of the schools, also approved the study. Each pupil was given a consent form to obtain informed parental consent. Informed assent was also sought from each pupil before inclusion in the study.

## 3 Results

### 3.1 Bacteria and fungi count in indoor classroom environment

The median TFC in wet season (70.0 CFU/m^3^) was higher than the counts in the dry season (48.33 CFU/m^3^). Likewise, the TBC in wet season (706.33 CFU/m^3^) was higher than that of dry season (419.17 CFU/m^3^) (*p* < 0.001). However, the TFC (in wet season) and TBC (in wet and dry seasons) exceeded the WHO guideline limit of ≤50 CFU/m^3^ for TFC and ≤500 CFU/m^3^ for TBC in an indoor environment.

### 3.2 Total bacterial and fungal counts across sampling locations

**Figure 2:**
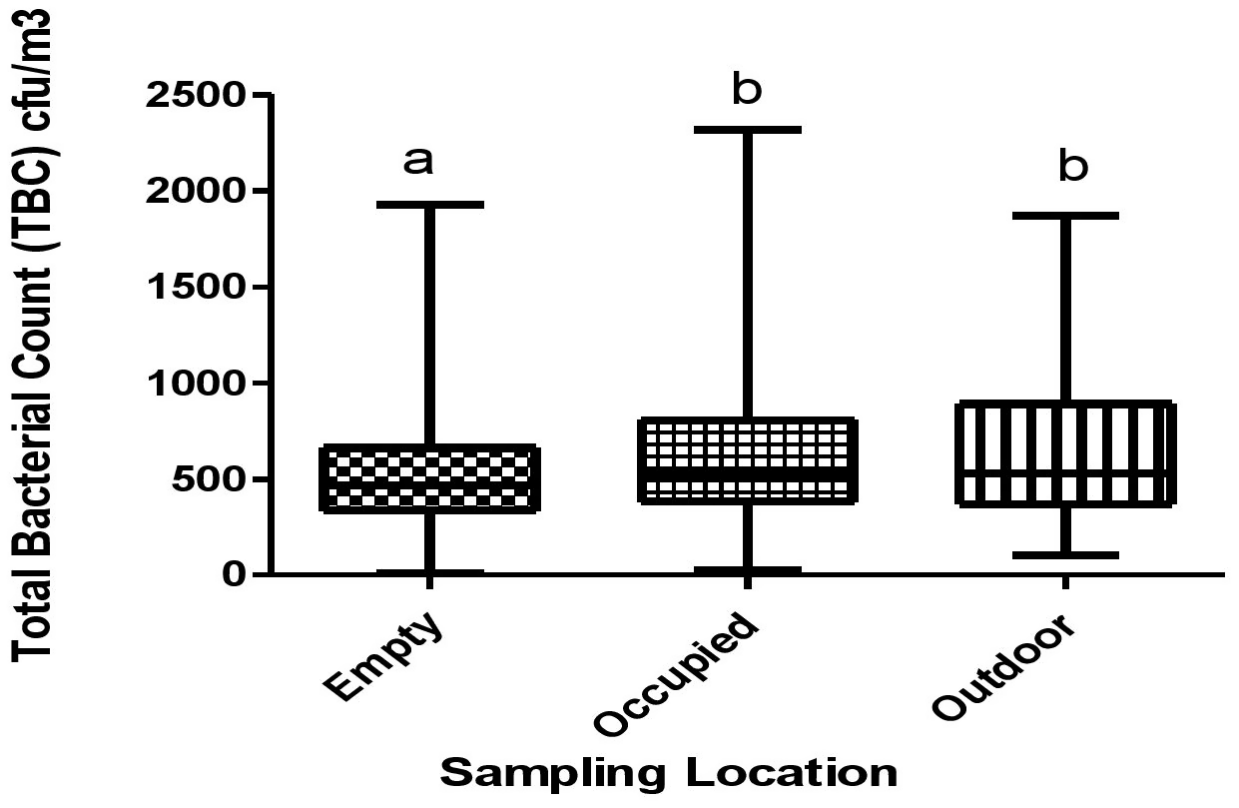
Total bacteria counts across sampling locations. Whisker box plots with non-similar alphabets are significantly different from each other (*p* < 0.05).

Figure 2 shows the distribution of the median TFC across sampling locations. The median TBC were 527.0 CFU/m^3^, 467.0 CFU/m^3^, and 526.0 CFU/m^3^ in the outdoor, empty classrooms and occupied classrooms, respectively. There was a significant difference between empty and occupied classrooms, and between empty classrooms and outdoor (*p* < 0.05).

**Figure 3:**
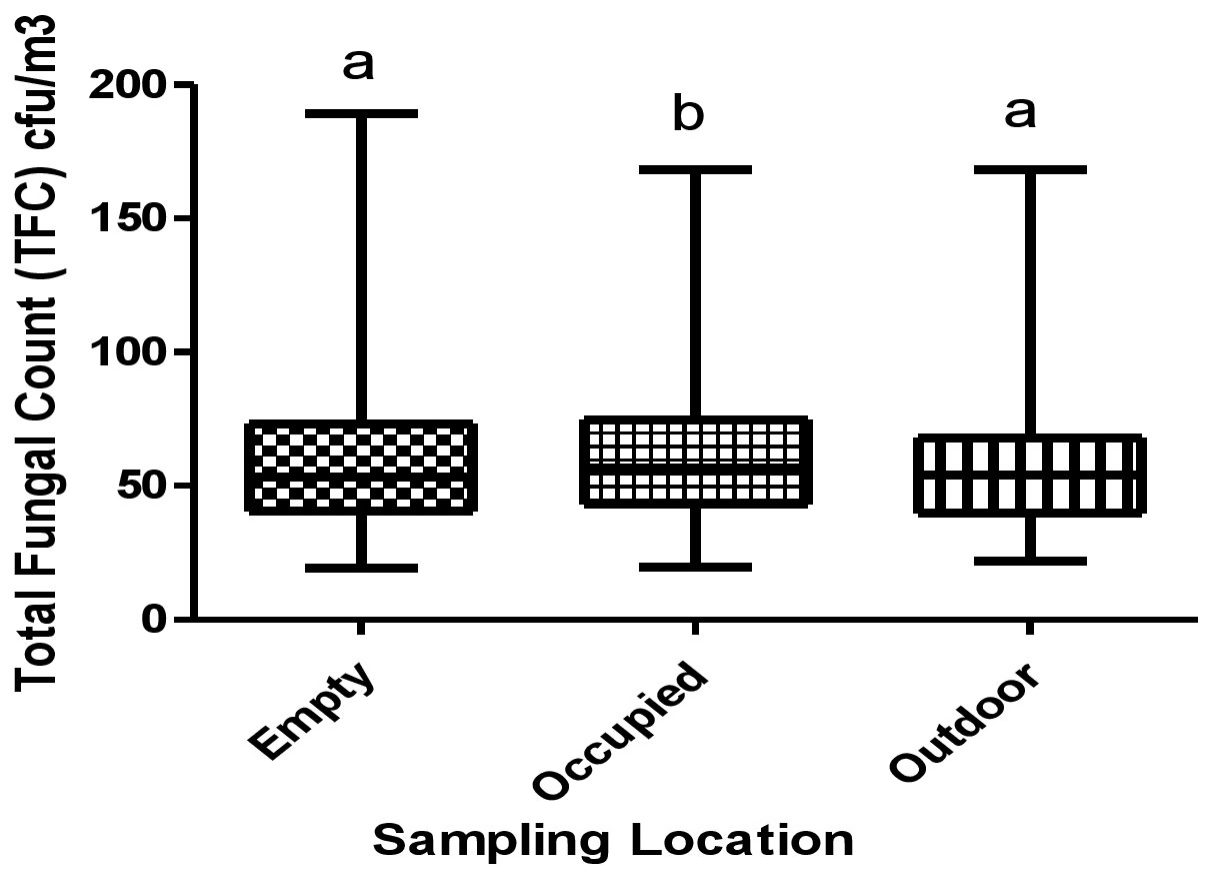
Total fungal counts across sampling locations. Whisker box plots with non-similar alphabets are significantly different from each other (*p* < 0.05).

The median TFC in outdoor, empty classrooms and occupied classrooms were 54.0 CFU/m^3^, 53.0 CFU/m^3^, and 57.0 CFU/m^3^, respectively (Figure 3). The 25 and 75% percentiles of TFC were 40.0 and 68.0 CFU/m^3^ for outdoor, 41.0 and 73.0 CFU/m^3^ for empty classrooms and 43.0 and 75.0 CFU/m^3^ for occupied classrooms. There was a significant difference between TFC in empty and occupied classrooms (*p* < 0.05), and also between occupied classrooms and outdoor (*p* < 0.05).

### 3.3 TFC and TBC across sampling locations during wet and dry seasons

**Figure 4:**
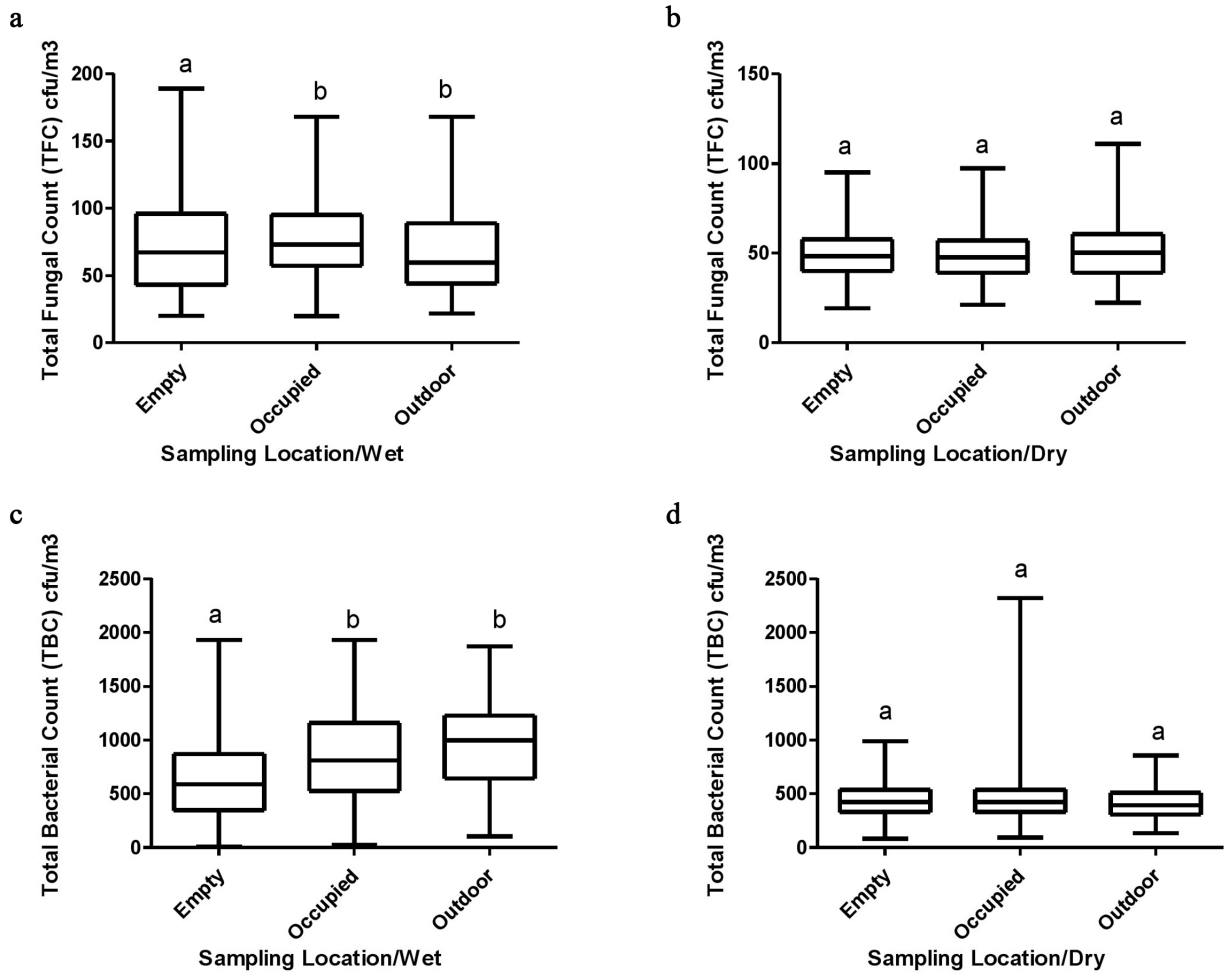
**(a–d)** TFC and TBC across sampling locations during wet and dry seasons. Whisker box plots with non-similar alphabets are significantly different from each other (*p* < 0.05).

In Figures 4a–d, the median TFC in outdoor, empty and occupied classrooms (60.0, 67.0, 73.0 CFU/m^3^) during the wet season were higher than the TFC in outdoor, empty and occupied classrooms (50.0, 48.0, 48.0 CFU/m^3^) during the dry season, respectively. In the wet season, the median concentration of TFC was highest when classrooms were occupied by pupils while it was highest in the outdoor environment during the dry season. There was a significant difference in the levels of TFC in the outdoor environment and empty classrooms, and empty and occupied classrooms during wet season.

Also, the median TBC in outdoor, empty and occupied classrooms (997.0, 589.0, 811.0 CFU/m^3^) were highest in the wet season than the median TBC in outdoor, empty and occupied classrooms (393.0, 424.0, 423.0.0 CFU/m^3^) in the dry season, respectively (Figures 4a–d). The median TBC was highest in the outdoor environment in the wet season while the highest mean TBC was recorded in occupied classrooms in the dry season. The median TBC across all sampling locations in the wet season exceeded the WHO recommended guideline of ≤500 CFU/m^3^ for an indoor environment. There was a significant difference in the levels of TBC in the outdoor environment and empty classrooms, and empty and occupied classrooms during the wet season. No statistical difference was observed in the levels of TFC and TBC between the sample locations during the dry season.

### 3.4 TFC, TBC and sampling time in sampled schools

The TFC in the morning hours (54.00 CFU/m^3^) was not statistically different from the TFC in the afternoon hours (54.67 CFU/m^3^). Higher TBC was recorded in the afternoon hours (510.00 CFU/m^3^) than in the morning hours (489.67 CFU/m^3^) (*p* = 0.009). The observed TFC in the morning and afternoon exceeded the WHO guideline limit of ≤50 CFU/m^3^ for an indoor environment, while the TBC in the afternoon hours exceeded the standard limit of ≤500 CFU/m^3^ in an indoor environment.

### 3.5 Size distribution of bacteria and fungi

In the wet season, the dominant median bacterial fraction (21.7%) was recorded on the fourth stage (2.1–3.3 μm), and the least (11.6%) on the sixth (0.65–1.1 μm) stage. During the dry season, airborne bacteria aerosols presented maximum concentration (20.3%) on the second stage (4.7–7.0 μm) stage and the minimum (12.2%) on the fifth (1.1–2.1 μm). Statistically, there was a significant difference in airborne bacteria was observed among all the stages in dry and wet seasons (*p* < 0.001).

The highest proportion of fungi in the wet season was recorded in the size range 2.1–3.3 μm and 0.65–1.1 μm (stages four and six) with fraction of 18.5%, respectively. The least proportion was found in the size range 1.1–2.1 μm (stage 5) with a fraction of 13.9%. In contrast, in the dry season, the highest (17.6%) and lowest (15.6%) fungi concentrations were found at the size fractions 1.1–2.1 μm and 2.1–3.3 μm, respectively. A significant statistical difference in fungi levels was observed among all the stages in dry and wet seasons (*p* < 0.001).

### 3.6 Fungal and bacterial respirable fractions during wet and dry seasons

The respirable airborne bacterial and fungal counts (sum of the third stage to the sixth stage) in the wet and dry seasons are summarized in Table 2. The fungal respirable fraction (FRF) and the bacterial respirable fraction (BRF) in the wet season (283.00 and 2,890.00 CFU/m^3^) were higher than the FRF and BRF in the dry season (196.00 and 1,661.00 CFU/m^3^), respectively.

**Table 2:** Fungal and bacterial respirable fractions during wet and dry seasons.

BC	Wet				Dry			
	GM (95% CI)	Median (IR)	Min	Max	GM (95% CI)	Median (IR)	Min	Max
FRF	300.29 (292.62–307.95)	283.00 (207.50)	42.00	764.00	203.14 (199.62–206.65)	196.00 (94.00)	68.00	516.00
BRF	3,227.91 (3,134.39–3,321.45)	2,890.00 (2,628.50)	40.00	7,712.00	1,740.64 (1,700.09–1,781.20)	1,661.00 (1,045.00)	158.00	13,062.00

### 3.7 Fungal and bacterial respirable fractions and sample location

Table 3 illustrates the median concentration of FRF and BRF across sampling locations. The median FRF was 217.00, 214.00 and 230.00 CFU/m^3^ in the outdoor, empty classrooms and occupied classrooms, respectively. Moreover, the median BFR was 3,164.00 CFU/m^3^, 3,232.23 CFU/m^3^, and 3,906.92 CFU/m^3^ in the outdoor, empty classrooms and occupied classrooms, respectively. Higher proportion of FRF and BRF was recorded in occupied classrooms.

**Table 3:** Fungal and bacterial respirable fractions and sample locations.

BC	Outdoor		Empty		Occupied		*p*-value
	GM (95% CI)	Median (IR)	GM (95% CI)	Median (IR)	GM (95% CI)	Median (IR)	
FRF	240.19 (228.54–251.84)	217.00 (144.50)	246.67 (239.60–253.75)	214.00 (150.00)	253.68 (247.21–260.14)	230.00 (144.00)	0.061
BRF	3,908.45 (3,681.54–4,135.36)	3,164.00 (3,139.50)	3,232.23 (3,126.85–3,337.62)	2,800.00 (1,954.00)	3,906.92 (3,777.65–4,036.20)	3,906.92 (2,544.00)	<0.001

### 3.8 Estimation of dose rates

The estimated inhaled doses of bacteria and fungi in wet and dry seasons are presented in Table 4. In the wet seasons, children and adults inhaled larger dosages of bacterial and fungal spores than in the dry seasons. Furthermore, children inhaled higher levels of bacterial and fungal aerosols in the air than adults. The ADD of children in the wet and dry seasons (6.67 × 10^2^ and 3.10 × 10^2^ CFU/Kg/day) were two times higher than that of adults (3.48 × 10^2^ and 1.61 × 10^2^ CFU/Kg/day).

**Table 4:** Dose rates (CFU/Kg/day) of total bacteria and fungi aerosols in occupied classrooms.

Population	TBC		TFC	
	Wet	Dry	Wet	Dry
Children	6.67 × 10^2^	3.48 × 10^2^	6.00 × 10^1^	3.95 × 10^1^
Adult	3.10 × 10^2^	1.61 × 10^2^	2.79 × 10^1^	1.83 × 10^1^

### 3.9 Pupil socio-demographic characteristics and prevalence of respiratory symptoms

The pupils’ average age was 10.23 + 1.31 years. More than half (53.6%) of the pupils were females and in class six (67.0%). Also, more than half (55.2%) of the pupils have mothers whose highest educational level was secondary school, ditto for father’s education (54.5%) and mother’s occupation (57.9%) (Table 5).

**Table 5:** Socio-demographic characteristics of pupils.

Variable	Total	%
Age of pupils (years)		
≤10	221	39.9
>10	333	60.1
Gender		
Male	257	46.4
Female	297	53.6
Class of student		
Four	64	11.6
Five	119	21.5
Six	371	67.0
Father’s occupation		
Self-employed	235	42.4
Trading	126	22.7
Civil servant	114	20.6
Farming	79	14.3
Mother’s occupation		
Trading	321	57.9
Self-employed	140	25.3
Civil servant	60	10.8
Farming	33	6.0
Father’s educational level		
None	30	5.4
Primary	68	12.3
Secondary	302	54.5
Tertiary	154	27.8
Mother’s educational level		
None	30	5.4
Primary	81	14.6
Secondary	306	55.2
Tertiary	137	24.7

The overall prevalence of rhinitis ever, current rhinitis, wheeze ever and current wheeze were 41.9, 40.4, 47.4 and 35.9%, respectively Pupils who were males and ≤10 years experienced higher prevalence of rhinitis ever (57.4 and 54.6%), current rhinitis (51.5 and 50.9%) and current wheeze (43.4 and 43.4%), respectively. More than half, of pupils with rhinitis ever (55.2%) and wheeze ever (54.3%) have fathers who are self-employed. Pupils whose mothers were farmers recorded higher prevalence of rhinitis ever (70.0%), current rhinitis (66.7%), wheeze ever (63.3%) and current wheeze (63.3%). The overall prevalence of rhinitis ever, current rhinitis, wheeze ever and current wheeze were 41.9, 40.4, 47.4 and 35.9%, respectively (Table 6).

**Table 6:** Relationship between socio-demographic characteristics and reported respiratory morbidities among pupils.

Variable	Total	Rhinitis ever			Current rhinitis			Wheeze ever			Current wheeze		
		%	*χ*^2^-value	*p*-value	*N* (%)	*χ*^2^-value	*p*-value	*N* (%)	*χ*^2^-value	*p*-value	*N* (%)	*χ*^2^-value	*p*-value
Age of pupils (years)			0.172	0.678		1.211	0.271		0.499	0.480		0.057	0.811
≤10	205	54.6			54.1			49.8			43.4		
>10	307	52.8			49.2			52.9			42.3		
Gender			2.698	0.100		0.018	0.895		<0.001	0.985		0.071	0.790
Male	235	57.4			51.5			51.7			43.4		
Female	277	50.2			50.9			51.6			42.2		
Class of student			1.679	0.432		0.913	0.633		2.322	0.313		3.064	0.216
Four	59	45.8			47.5			49.2			32.2		
Five	117	55.6			48.7			46.2			43.6		
Six	336	54.2			52.7			54.0			44.3		
Father’s occupation			0.677	0.879		5.126	0.163		2.567	0.463		3.499	0.321
Trading	115	52.2			42.6			50.9			39.1		
Farming	70	50.0			55.7			54.3			42.9		
Civil servant	104	53.8			50.0			45.2			37.5		
Self-employed	223	55.2			54.7			54.3			47.1		
Mother’s occupation			5.133	0.162		5.079	0.166		2.078	0.556			
Trading	299	54.2			50.2			51.3			41.5	7.230	0.065
Farming	30	70.0			66.7			63.3			63.3		
Civil servant	53	54.7			58.5			47.2			49.1		
Self-employed	130	47.7			46.9			51.5			38.5		
Father’s educ. level			3.263	0.353		3.179	3.65		2.985	0.394		2.013	0.570
None	28	60.7			39.3			42.9			50.0		
Primary	63	61.9			54.0			50.8			42.9		
Secondary	280	52.9			53.6			54.8			44.3		
Tertiary	141	49.6			47.5			47.5			38.3		
Mother’s educ. level		3.974	3.974	0.264	6.408	6.408	0.093		1.532	0.675		0.100	0.992
None	28	60.7			28.6			53.6			42.9		
Primary	73	57.5			54.8			50.7			41.1		
Secondary	286	49.7			51.4			53.7			43.0		
Tertiary	125	58.4			53.6			47.2			43.2		

### 3.10 Unadjusted and adjusted odds ratio of FRF and BRF and other factors influencing rhinitis ever

The relationship between indoor FRF, BRF and rhinitis ever is presented in Table 7. At the unadjusted level of analysis, the odds of rhinitis ever were significantly higher among children whose classroom’s FRF was ≥50 (OR = 3.06, CI: 2.01–4.67, *p* < 0.001) and whose classroom’s BRF (OR = 3.12, CI: 0.06–4.73, *p < 0.001*) were higher than ≥500.

**Table 7:** Unadjusted and adjusted odds ratio of FRF and BRF and covariates of rhinitis ever.

Characteristics	Unadjusted estimates			Adjusted estimates		
	OR	95%CI	*p*-value	aOR	95%CI	*p*-value
FRF						
<50	1	–		1	–	
≥50	3.06	^*^2.01–4.67	<0.001	2.12	^*^1.30–3.47	0.003
BRF						
<500	1	–		1		
≥500	3.12	^*^2.06–4.73	<0.001	2.20	^*^1.35–3.59	0.002
Age						
<10	1			1		
≥10	0.93	0.65–1.32	0.678	0.92	0.63–1.36	0.686
Gender						
Female	1			1		
Male	0.75	0.53–1.06	0.100	1.51	^*^1.03–2.20	0.034
Cooking fuel type						
Clean	1	–		1		
Unclean	1.29	0.89–1.58	0.177	1.15	0.77–1.73	0.498

* Significant at 5%.

FRF, Fungi respirable fractions; BRF, Bacterial respirable fractions.

At the adjusted level of analysis, FRF and BRF were significantly associated with rhinitis ever even when other covariates were controlled for. For instance, the odds of having rhinitis ever were 2.12 (CI: 1.30–3.47, *p* = 0.003) and 2.20 (CI: 1.35–3.59, *p* = 0.002) higher among pupils who occupied classrooms with greater than the median score for FRF and BRF, respectively. The identified predictors of rhinitis ever are; being a male child (aOR = 1.51, CI: 1.03–2.20, *p =* 0.034) and living a house cooking with unclean fuel (aOR = 1.15, CI: 0.77–1.73, *p =* 0.498).

Tables 8–10 summarizes the results of the unadjusted and adjusted logistic regression for current rhinitis, wheeze ever and current wheeze, respectively. At the unadjusted level of analysis, exposure to high FRF (≥50 cfu/m^3^) at school was significantly associated with increased likelihood of having current rhinitis (OR = 2.73, CI: 1.81–4.13, *p* < 0.001), wheeze ever (OR = 1.74, CI: 0.16–2.61, *p* < 0.001) and current wheeze (OR = 2.88, CI: 1.81–4.59, *p* < 0.001). Moreover, exposure to high BFR increased the likelihood of having current rhinitis (OR = 2.69, CI: 1.80–4.04, *p* < 0.001), wheeze ever (OR = 2.32, CI: 1.54–3.51, *p* < 0.001) and current wheeze (OR = 6.99, CI: 3.84–12.72, *p* < 0.001).

**Table 8:** Unadjusted and adjusted odds ratio of FRF, BRF and covariates of current rhinitis.

Characteristics	Unadjusted estimates			Adjusted estimates		
	OR	95%CI	*p*-value	aOR	95%CI	*p*-value
FRF						
<50	1	–		1	–	
≥50	2.73	*1.81–4.13	<0.001	1.83	*1.14–2.93	0.012
BRF						
<500	1	–		1		
≥500	2.69	*1.80–4.04	<0.001	1.78	*1.11–2.85	0.016
Age						
<10	1			1		
≥10	0.82	0.58–1.17	0.271	0.81	0.56–1.18	0.637
Gender						
Female	1			1		
Male	0.98	0.69–1.38	0.895	1.09	0.76–1.58	0.637
Cooking fuel type						
Clean	1	–		1		
Unclean	1.11	0.77–1.60	0.567	1.03	0.69–1.54	0.872

“*” means significant values at *p*<0.05.

**Table 9:** Unadjusted and adjusted odds ratio of FRF, BRF and covariates of wheeze ever.

Characteristics	Unadjusted estimates			Adjusted estimates		
	OR	95%CI	*p*-value	aOR	95%CI	*p*-value
FRF						
<50	1	–		1	–	
≥50	1.74	*1.16–2.61	0.007	1.38	1.87–2.19	0.001
BRF						
<500	1	–		1		
≥500	2.32	*1.54–3.51	<0.001	1.93	*1.21–3.08	0.006
Age						
<10	1			1		
≥10	1.14	0.80–1.62	0.480	1.08	0.75–1.56	0.678
Gender						
Female	1			1		
Male	1.00	0.0.70–1.41	0.985	1.00	0.70–1.44	0.988
Cooking fuel type						
Clean	1	–		1		
Unclean	1.15	0.80–1.65	0.462	0.94	0.63–1.39	0.936

“*” means significant values at *p*<0.05.

**Table 10:** Unadjusted and adjusted odds ratio of respirable fungal and bacterial counts and other factors influencing current wheeze.

Characteristics	Unadjusted estimates			Adjusted estimates		
	OR	95%CI	*p*-value	aOR	95%CI	*p*-value
FRF						
<50	1	–		1	–	
≥50	2.88	*1.81–4.59	<0.001	1.88	*1.18–3.00	0.008
BRF						
<500	1	–		1		
≥500	6.99	*3.84–12.72	<0.001	2.77	*1.73–4.43	<0.001
Age						
<10	1			1		
≥10	0.96	0.67–1.37	0.811	0.86	0.59–1.27	0.678
Gender						
Female	1			1		
Male	0.95	0.67–1.36	0.790	1.11	0.76–1.62	0.584
Cooking fuel type						
Clean	1	–		1		
Unclean	1.04	0.72–1.50	0.831	0.85	0.56–1.28	0.435

“*” means significant values at *p*<0.05.

At the adjusted level of analysis, FRF and BRF were significantly associated with current rhinitis (aOR = 1.83, CI: 1.14–2.93, *p < 0.001*, aOR = 1.78, CI: 1.11–2.85, *p* < 0.001), wheeze ever (aOR = 1.38, CI: 0.87–2.19, *p* = 0.166, OR = 1.93, CI: 1.21–3.08, *p* = 0.006) and current wheeze (aOR = 1.88, CI: 1.18–3.00, *p =* 0.008, aOR = 2.77, CI: 1.73–4.43, *p* < 0.001), respectively when covariates were adjusted for.

## 4 Discussion

### 4.1 Levels of TBC and TFC in indoor classroom environment

In this study, the median TFC and TBC in wet season was significantly higher than the TFC and TBC in dry season. The TFC (in wet season) and TBC (in wet and dry seasons) exceeded the WHO guideline limit. Pupils who spend long hours within these settings may be unduly exposed to the burden of bioaerosols present in such environment across different seasons. The high burden of bioaerosols recorded in wet season may account for the high prevalence of respiratory outcomes that usually characterize this season (24).

### 4.2 TBC and TFC in empty and occupied classrooms

This study shows that the TBC and TFC in occupied classrooms were higher than the TBC and TFC in empty classrooms. The difference in observed concentrations between occupied and unoccupied classrooms was probably caused by variations in room use patterns, student density, and children’s activity. Children’s activity, conversation, and material interaction during school hours might resuspend settled particles and increase the amount of bioaerosols’ shedding from surfaces, clothing, and skin. Studies have reported that the presence of humans in an indoor environment are the major sources of bioaerosols in built environment (25). Humans harbor 10^12^ microbes on their skin, 10^14^ microbes in their respiratory tract (26), have an emission rate of ~30 mg per person hour, corresponding to 3.7 × 10^7^ and 7.3 × 10^6^ bacterial and fungal genome copies and sheds 10^7^ bacteria per person in an hour (27). Bioaerosols are released from humans into the indoor air during respiration, shedding of the cells of the skin, sweating, and repeated movement resulting in particle resuspension (27).

### 4.3 Size distribution of bacteria and fungi

Findings from this study show variations in the distribution of bacteria and fungi counts across the six-stages of the impactor during the wet and dry seasons. The dominant bacteria fraction in the wet season was recorded at the aerodynamic size fractions of 2.1–3.3 μm (stage 4) while the dominant fungi fraction was recorded in the size range 2.1–3.3 μm and 0.65–1.1 μm (stages four and six). In the dry season, there was variation in the distribution of bioaerosols with the maximum fractions being of the aerodynamic diameter 4.7–7.0 μm and 1.1–3.3 μm, respectively.

Different microorganisms have varying aerodynamic sizes ranging from about 0.02–100 μm (23). The aerodynamic diameter (AD) determines their survival, period of aerosolisation in the air and site of deposition within the human respiratory system and the corresponding health impact in human (28, 29). Bacterial cells occur as single cells or small aggregates, typically falling within smaller particle size ranges (respirable fractions). In contrast, fungal spores vary widely in size depending on species—some (e.g., *Aspergillus*, *Penicillium*) produce smaller spores that are captured in lower stages, while others (e.g., *Alternaria*, *Cladosporium*) produce larger spores more likely to be collected at upper stages.

Moreover, this current study shows that a statistically significant difference exists between the bacteria fractions recorded across all the stages in dry and wet seasons (*p* < 0.001). This was also true for the fungal fractions. These findings were consistent with that of Li et al. (30), who also reported seasonal and regional variations in the size distribution of bioaerosols. Seasonal environmental factors are important in determining how bacteria and fungus proliferate, sporulate, and aerosolize. Higher temperatures and humidity during the rainy season foster microbial growth and active sporulation on interior surfaces, increasing the number of larger and more viable spores in the atmosphere. This usually leads to a more widespread dispersion of bioaerosols throughout the different impactor stages, including the upper stages that absorb bigger particles (30). Nonetheless, some studies have reported no significant difference among the size distributions of bacteria and fungi under different weather conditions (11, 30).

Furthermore, the findings from this study show that the respirable bacterial fractions and the fungi fractions in the wet season were higher than those in the dry season. This translates to about 67.5% of the total bacterial and 77.8% of the fungal aerosols being of the respirable size fractions (aerodynamic diameter of 1.1–4.7 μm), which corresponds to the region between the human bronchus and the alveolar duct (lower respiratory tract) (31). The presence of these bioaerosols in the human bronchus and the alveolar duct are of great concern because of the absence of cilia in these airways. These bioaerosols can be resident within the lungs for longer periods except if they are degraded or removed with the help of pulmonary macrophages (32).

Within the human bronchus and the alveolar duct, bioaerosols can cause allergic alveolitis and other adverse health outcomes (33). Bioaerosols deposited in the upper airways can trigger some allergic or inflammatory response (e.g., rhinitis) and may induce lung diseases in susceptible populations (32). For instance, some bioaerosols including *Aspergillus* spp., *Bacillus* spp., and *Mycobacterium* spp. are capable of causing pulmonary infection if they penetrate deeper into the smaller air ways (32). With more than 60% of the TBC and TFC in this study being of the respirable size fraction, the indoor air quality in the sampled schools may pose a significant threat to the health of the pupils.

The concentrations of respirable bioaerosols are greatly influenced by meteorological factors including temperature, humidity, and rainfall; in the research area, there are noticeable seasonal differences between the wet and dry seasons. The development and sporulation of bacteria and fungus on interior surfaces and in the surrounding environment are facilitated by high humidity and increased surface moisture during the rainy season, which may result in larger concentrations of airborne microbial particles. On the other hand, during the dry season, especially in areas with inadequate ventilation or high human activity, decreased humidity and higher dust levels may promote the resuspension of deposited spores and particulate matter into the indoor air.

### 4.4 Average daily absorbed dose of bioaerosols

The findings from this study show that children breathe in higher doses of bioaerosols in wet and dry seasons (667 and 310 CFU/Kg/day) than adults in wet and dry seasons (348 and 161 CFU/Kg/day). The absorbed dose in children were up to two times higher than in adults. This position has been reported in similar studies (13, 34). Bragoszewska et al. (23), reported that bacterial inhaled dose in children ranged between 545.8 and 929.9 CFU/Kg/day, and ranged between 272.6 and 331.9 CFU/Kg/day in adults. The inhalation rates of children differ significantly from that of adults because of their physiology, size and activity level. Children have weaker defenses against air pollutants than adults, have a lower ability to metabolize and detoxify environmental agents, and have a more porous airway epithelium to inhaled air pollutants (35). They possess a greater oxygen intake rate per unit body weight than adults due to their rapidly growing rate and the large surface area of their lungs. This implies that the volume of air passing through the lungs of a child is twice that of adults.

Although the cross-sectional design makes it difficult to assess causality, the correlation between greater absorbed doses and respiratory symptoms is consistent with other research that shows indoor bioaerosol exposure is associated with poor respiratory outcomes, particularly in children. In line with other research showing a dose–response relationship, our results lend biological credence to the fact that microbial exposure contributes to respiratory morbidity.

### 4.5 Socio-demographic characteristics and respiratory symptoms

The overall prevalence of rhinitis ever, current rhinitis, wheeze ever and current wheeze in this study were 41.9, 40.4, 47.4, and 35.9%, respectively. According to a report from different centers across the globe that took part in the ISAAC Phase III study, the global average for present wheeze in 13–14 year old children is 14% (36). Differences in prevailing weather conditions, geographical locations, indoor air quality, size distribution and concentration of bioaerosols may explain the marked differences in the prevalence of respiratory symptoms across countries.

Individual personal characteristics such as age and gender play a significant role in the occurrence of respiratory disorders (37). Findings from this study show that pupils who were males and ≤10 years experienced higher prevalence of rhinitis ever, current rhinitis, wheeze ever and current wheeze. The United Nations had reported that the natural environment in developing countries favors female survival (38). The reason being that male infants possess some biological characteristics that make them less likely to live than female infants. The influence of the x-linked immunoregulatory genes, which provides females better resistance to infection, is one of these characteristics (39, 40). According to Waldron et al. (39), there is a danger of lung immaturity in males as a result of testosterone’s action on the lungs, which predisposes them to respiratory distress syndrome. There is also a difference in surfactant synthesis, the size of the airway, and airway resistance between male and female children (41). This makes male children to be more vulnerable to childhood respiratory illnesses due to these sex differences.

Higher prevalence of respiratory symptoms among pupils whose mothers were farmers was documented in this study. Since different occupations involve varied levels of environmental exposure, occupational activities are expected to affect indoor levels of bacterial and fungal spores in the respirable fraction. People who work in agriculture, for instance, are exposed to organic dust, dirt, decomposing vegetation, and animal waste—all of which are known to contain significant concentrations of microbial spores. In settings where work and home spaces frequently overlap, these bioaerosols may enter the home through contaminated clothing and tools.

Evidence presented in literature suggests that prenatal air pollution exposure is linked to the occurrence of respiratory and immune symptoms in their children, which can lead to respiratory morbidity and allergic reactions later in life (42). Rural school-aged children living on and off farms in Canada were found to have a link between farming activities and respiratory health in related studies (43). In children and adults, these exposures have been related to asthma, rhinitis, chronic bronchitis and decreased forced expiratory volume (FEV1) (44). However, instances of reduction in the incidence of allergic respiratory morbidities among farmers’ children have been reported (45, 46).

### 4.6 Predictors of respiratory allergic symptoms in school pupils

Furthermore, the findings from this study show that at the adjusted level of analysis, fungi and bacteria within the respirable size fractions were significantly associated with rhinitis ever, current rhinitis, wheeze ever and current wheeze. This clearly shows that the aforementioned risk factors are predictors of the observed respiratory symptoms.

The size fractions of fungal and bacterial species determine their deposition site within the human respiratory tract. The size distribution of indoor airborne bioaerosols and the accompanying deposition site within the lungs or airways establish the association between exposure to these bioaerosols and probable health effects (9). For instance, fungal spores such as *Penicillium brevicompactum, Aspergillus fumigatus, Cladosporium macrocarpum*, and *Aspergillus niger*, with a diameter of less than 10 μm, can penetrate into the bronchi and trigger allergic responses of the lower respiratory tract (32, 47).

## 5 Study limitations

A significant drawback of this research is that wheeze and rhinitis were analyzed independently using the ISAAC questionnaire framework, which made it impossible to evaluate their co-occurrence or synergistic effects. The study only used validated but self-reported symptom data, which made it harder to support causal inference. Additionally, the lack of inflammatory biomarkers hindered understanding of underlying immunopathological pathways. However, given the limited data on indoor air quality and respiratory health in school settings within our context, this research contributes to filling a critical knowledge gap and lays the foundation for future longitudinal or intervention-based studies that can further clarify causality and mechanisms.

## 6 Conclusion

This study provides key information about the spatial and temporal trends of indoor bioaerosol exposure in educational settings and how they can relate to respiratory symptoms in pupils. We were able to characterize bioaerosol size distribution, concentrations and types that can affect respiratory health outcomes by gathering data across seasons and analyzing bioaerosols size distributions.

The levels of bacterial and fungal spores of respirable fractions observed varied greatly in terms of season and occupancy with higher concentrations recorded in occupied classrooms and during the wet season. These levels exceeded the recommended guideline limits which implies a higher adverse health risk for pupils.

Moreover, variations in the distribution in the levels of bacteria and fungi across the six-stages of the impactor during the wet and dry seasons was reported in this study. A greater proportion of the bacterial and fungal aerosols were of the respirable size fractions which corresponds to the region between the human bronchus and the alveolar duct (lower respiratory tract). The presence of these bioaerosols in the human bronchus and the alveolar duct are of great concern because of the absence of cilia in these airways.

In addition, children have higher tendency to inhaled higher bacterial and fungal aerosols than adults. The absorbed dose in children were up to two times higher than in adults. These shows that school environment is an important location for children’s exposure to biological and chemical contaminants, given their high level of vulnerability. The highly absorbed dose of bioaerosols among the pupils may explain the increased prevalence of respiratory outcomes documented in this study.

Moreover, the study integrated health data based on symptoms using the validated ISAAC questionnaire, which enabled us to investigate correlations between exposure indicators and reported respiratory symptoms. Although causal inference is limited by the study’s cross-sectional design, the discovered connections provide significant epidemiological evidence of possible risk links in an understudied, real-world population.

Improvement in indoor sanitary conditions and hygiene practices are key to dipping the levels of indoor bioaerosols fractions and consequently, reduction in the prevalence of associated respiratory morbidities. While dry sweeping should be avoided, wet cleaning techniques and regular surface disinfection help reduce the resuspension of settled particles. Encouraging handwashing and respiratory etiquette, as well as providing access to clean water, soap, and hand sanitizers, is still a low-cost but efficient way to promote hygiene.

## Data Availability

The raw data supporting the conclusions of this article will be made available by the authors without undue reservation.
